# Targeting β-catenin in acute myeloid leukaemia: past, present, and future perspectives

**DOI:** 10.1042/BSR20211841

**Published:** 2022-04-21

**Authors:** Megan Wagstaff, Brandon Coke, Georgia R. Hodgkiss, Rhys G. Morgan

**Affiliations:** 1 School of Life Sciences, University of Sussex, Brighton, U.K.; 2 School of Biological Sciences, University of Southampton, Southampton, U.K.

**Keywords:** acute myeloid leukaemia, beta-catenin, small molecules, Wnt signalling

## Abstract

Acute myeloid leukaemia (AML) is an aggressive disease of the bone marrow with a poor prognosis. Evidence suggests long established chemotherapeutic regimens used to treat AML are reaching the limits of their efficacy, necessitating the urgent development of novel targeted therapies. Canonical Wnt signalling is an evolutionary conserved cascade heavily implicated in normal developmental and disease processes in humans. For over 15 years its been known that the central mediator of this pathway, β-catenin, is dysregulated in AML promoting the emergence, maintenance, and drug resistance of leukaemia stem cells. Yet, despite this knowledge, and subsequent studies demonstrating the therapeutic potential of targeting Wnt activity in haematological cancers, β-catenin inhibitors have not yet reached the clinic. The aim of this review is to summarise the current understanding regarding the role and mechanistic dysregulation of β-catenin in AML, and assess the therapeutic merit of pharmacologically targeting this molecule, drawing on lessons from other disease contexts.

## Introduction

Acute myeloid leukaemia (AML) is an aggressive and heterogeneous clonal disease of haematopoietic stem/progenitor cells (HSPC) in the bone marrow with approximately 3,200 cases annually in the UK (Cancer Research UK statistics). The disease predominates in older adults (60 years plus) and is characterised by the uncontrolled proliferation and arrested differentiation of myeloid committed blasts in the bone marrow. Patients experience the rapid onset of symptoms originating from impaired myelopoiesis including immunosuppression, defective haemostasis, and severe anaemia, and will ultimately succumb to bone marrow failure without timely medical intervention. Despite improvements in overall AML survival over the past 60 years, the prognosis for older patients, or those harbouring adverse gene mutations or cytogenetic events, remains poor [1]. With life expectancy rising across the developed world, the burden of AML on population health has grown proportionally and is set to grow further over the coming decades [2]. Therefore, there is an urgent need for novel, targeted, efficacious therapies that are well tolerated by patients and induce robust, long-term clinical remissions.

It’s an exciting time for AML treatment with an arsenal of novel agents recently approved by the Food and Drug Administration (FDA) and the European Medical Agency (EMA) for deployment against newly diagnosed, relapsed or refractory AML. Such therapies are gradually supplementing the dated, broad spectrum, toxic chemotherapies traditionally used to treat AML, instead targeting specific molecular aberrations relevant to the patient. These include midostaurin for FLT3 mutant AML, venetoclax for raised BCL2 AML, ivosidenib/enasidenib for IDH mutant AML or gemtuzumab ozogamicin for CD33^+^ AML [3,4]. As we usher in this new era of precision medicine, the importance of thoroughly characterising molecular abnormalities in leukaemia stem cells (LSC; the cells widely accepted to initiate, maintain, and re-establish AML) remains vital in the ongoing development of novel agents. One molecule heavily implicated in AML, but incompletely understood, is β-catenin: the central effector of Wnt signalling. This review will summarise the current understanding around β-catenin dysregulation in AML and the progress to date in therapeutically targeting this molecule in leukaemia.

## Canonical Wnt signalling

**Figure 1:**
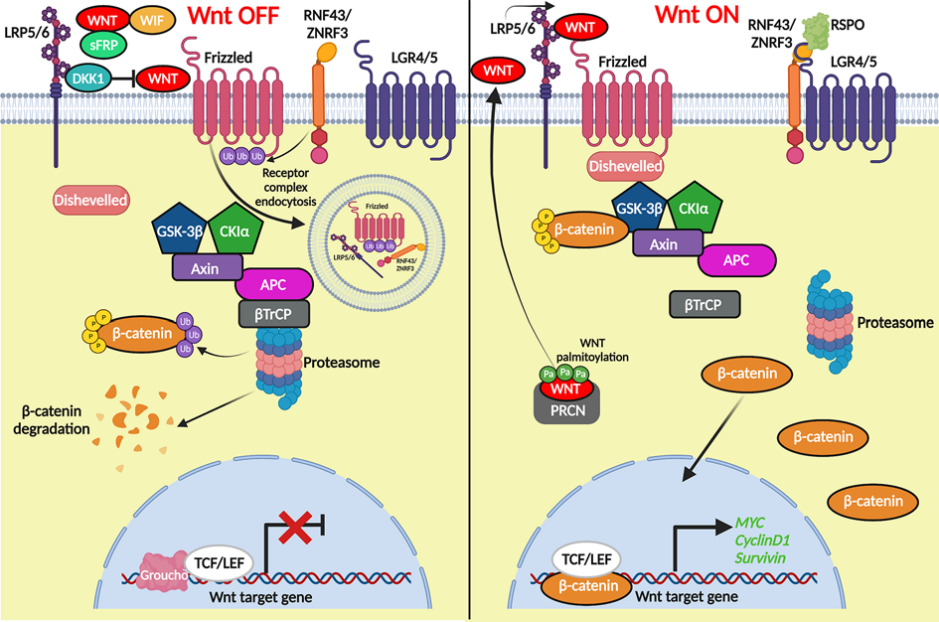
Outline of canonical Wnt signalling In the absence of a Wnt ligand bound to LRP and Frizzled receptors, β-catenin is bound by a destruction complex consisting of GSK3β, Axin, CK1 and APC. β-Catenin is phosphorylated leading to subsequent ubiquitination by βTrCP and degradation in the proteasome. Wnt target genes remain off as TCF is bound to the transcriptional repressor Groucho. Upon binding of a Wnt ligand to LRP/Frizzled the destruction complex is recruited to phosphorylated LRP through Axin where β-catenin is bound and phosphorylated. However, βTrCP can no longer ubiquitinate β-catenin causing saturation of the DC and subsequent stabilization of β-catenin which translocates to the nucleus, binds TCF/LEF, and activates Wnt target genes (created using Biorender).

The Wnt/β-catenin pathway is an evolutionary conserved signal transduction cascade with important roles in normal human development and disease. In particular, canonical Wnt signalling has critical roles in tissue homeostasis and the regulation of adult stem cells in several organ systems including the colon, skin, liver and mammary gland [5]. In the absence of secreted Wnt ligands bound to the LRP/Frizzled receptors, the central mediator β-catenin is bound by a destruction complex (DC) consisting of adenomatous polyposis coli (APC), axin, casein kinase-1 (CK1) and glycogen synthase kinase-3 (GSK3β) (Figure 1). Sequential phosphorylation of the β-catenin molecule then takes place, first on Ser45 by CK1, then on Thr41, Ser37 and Ser33 by GSK3β creating a docking site for βTrCP to induce ubiquitination and proteasomal degradation. The constitutive degradation of β-catenin prevents its nuclear accumulation and ability to serve as a co-factor for T-cell factor (TCF)/lymphoid enhancer factor (LEF) mediated transcription, leaving them Groucho bound and Wnt target genes repressed.

Upon appropriate binding of a Wnt ligand to the LRP/Frizzled receptors, the DC is relocalised with dishevelled (DVL) to the membrane where β-catenin remains bound and phosphorylated. However, βTrCP can no longer ubiquitinate β-catenin leaving the DC saturated and newly synthesised β-catenin free to accumulate in the cytosol. From here, β-catenin eventually translocates into the nucleus, through a variety of context-dependent mechanisms [6], where it displaces Groucho and binds TCF/LEF to activate Wnt target genes such as *MYC* [7], *BIRC5* [8], and *CCND1* [9]. In addition, there is a considerable amount of context dependent ‘fine tuning’ built into the system to ensure the appropriate dose and duration of Wnt signalling in the correct tissue at the right time. These include the presence of transmembrane E3 ubiquitin ligases such as RNF43/ZNRF3 controlling Wnt receptor turnover [10], the secretion of Wnt antagonists such as dickkopf (DKK), Wnt inhibitory factor (WIF) or secreted FZD-related proteins (sFRP) which either block Wnt receptors or sequester Wnts away from targets [11], or the presence of dominant negative isoforms of TCF/LEF which may bind DNA but lack the β-catenin binding domains necessary to transduce a Wnt signal [12]. Finally, the Wnt pathway can also mediate its own intensity through the activation of Wnt target genes that are themselves either positive (*LEF1*, *LGR5*, *FZD7*, *WNT3A*) or negative (*AXIN2, DKK1, SFRP2, RNF43/ZNRF3*) regulators of the pathway (https://web.stanford.edu/group/nusselab/cgi-bin/wnt/target_genes). All the above mechanisms ensure a ‘just right’ level of Wnt signalling (and β-catenin) is achieved that is both safe and appropriate for normal biological demand, but ‘just right’ Wnt dosages must also be optimal for tumourigenesis [13–15].

## The β-catenin molecule

β-Catenin is a member of the armadillo family of proteins which includes close homologues α-catenin (Alpha E-catenin), γ-catenin (plakoglobin), and δ-catenin (p120 catenin). Proteins of this family are typically characterised by a highly conserved central armadillo (ARM) domain of repeating ∼42 amino acid ARM motifs which contain three α-helices [16]. This central domain is responsible for binding most protein partners, which are highly varied and allow the proteins to participate in a diverse range of cellular functions from adhesion to transcription. β-Catenin contains a central ARM domain of 12 repeats flanked by an amino and carboxy terminus. The amino terminus contains the phospho-degron site which mediates the molecule’s stability, whilst the carboxyl terminus provides specificity and harbours a transactivation domain [17]. The β-catenin molecule lacks any canonical nuclear localisation signal (NLS) or nuclear export signal (NES), and instead nuclear entry/exit is facilitated by a host of NLS/NES containing chaperone proteins that act in a tissue-dependent manner [6]. As above, β-catenin’s structure allows it to interact with a diverse repertoire of proteins which has led to its designation as a dual function protein in adherence and signal transduction. Any free β-catenin in the cell typically ends up in adherens junctions bound to cadherins (and other catenins) where it mediates tight homotypic cell adhesion. However, whether β-catenin performs such a function in blood remains unresolved as such strong cell adhesion structures are a key feature of epithelial tissues, and not characteristic of haematopoietic cells.

## β-Catenin in normal myelopoiesis

Characterising β-catenin’s role in normal HSC and myeloid development is a vital prerequisite for understanding its pathogenic role in leukaemia, however this has proved enigmatic and challenging.

From the late 1990s, it was demonstrated that secreted Wnt ligands such as WNT1, WNT2B, WNT3A and WNT10B (which ultimately stabilise β-catenin expression) were expressed in bone marrow stroma and capable of significantly expanding HSC numbers *in vitro* [18–20]. Subsequent studies showed that enforced expression of constitutively active β-catenin in HSCs inhibited myeloid differentiation whilst extending the survival and self-renewal capacity of HSC [21,22], whilst loss of β-catenin led to reduced long-term growth and maintenance of HSC [23]. Such findings suggest a role for β-catenin in promoting the maintenance and self-renewal of HSC, a role conserved across multiple adult stem cell types including the gut, breast, stomach, liver, and skin [5].

Recent studies have indicated that β-catenin’s influence in human myelopoiesis is not restricted to the stem cell compartment. Data from the Qian group showed that activated β-catenin can inhibit monocyte/macrophage differentiation by disrupting PU.1-mediated transcription [24], a key transcription factor controlling granulocyte/monocyte commitment also known to be regulated by γ-catenin [25]. Other studies have suggested β-catenin has a role in regulating important characteristics of mature monocytes such as adherence and migration [26,27]. A further role for β-catenin in myelopoiesis has recently been reported in normal and emergency granulopoiesis. Disruption of the β-catenin:TCF4 interaction (through expression of a dominant negative TCF4 mutant), resulted in impaired granulocytic differentiation driven by repressed G-CSFR expression and disrupted G-CSF signalling [28].

In contrast, other studies have shown that expression of a constitutively active form of β-catenin (lacking phosphorylation sites required for degradation) in murine HSCs leads to exhaustion of the HSC pool, impaired repopulating capacity, reduced survival, and disruption to multilineage differentiation potential [29–31]. Furthermore, multiple *in vivo* studies have demonstrated the knockdown of β-catenin and/or γ-catenin has no detrimental short or long term effects on HSCs, myelo- or indeed lymphopoiesis [32–35]. It’s important to note that in some of these models residual TCF activity remained, or was not checked, meaning Wnt signalling may not have been completely extinguished and we now know minimal Wnt signalling can sustain normal haematopoiesis (see below) [36]. Finally, modulation of upstream Wnt signalling components known to increase β-catenin stability have also produced contradictory results; inactivation of APC led to impaired HSC self-renewal potential [37,38], whilst PORCN deletion (controlling Wnt ligand secretion) was dispensable altogether for HSC self-renewal, proliferation and differentiation [39].

At first, these results appear confusing and are partly explained by the breath of experimental models and strategies used to investigate them. However, perhaps the most coherent explanation provided to date has highlighted the importance of Wnt signalling dose [15]. The spatiotemporal control of Wnt signalling is crucial to ensure the correct duration and amplitude of Wnt signalling (and β-catenin stability) during normal development. Elegant work from the Staal group using targeted hypomorphic alleles and a conditional deletion allele of APC, produced a gradient of five differing Wnt signaling levels *in vivo*. This model demonstrated that only very low levels of Wnt signalling (and β-catenin) were required to sustain normal HSCs with elevated levels observed during myeloid and T-cell development. Very high levels of Wnt signalling impaired HSC self-renewal and differentiation potential, in keeping with previous reports above. That such low levels of Wnt/β-catenin signalling are required for normal haematopoiesis makes β-catenin an attractive therapeutic target in AML since much of its overactivity could be safely eliminated with minimal myelotoxicity.

## β-Catenin in AML

Over the past twenty years a plethora of studies have demonstrated dysregulated Wnt/β-catenin signalling in AML which can be broadly split into cell intrinsic and extrinsic influences.

### Cell intrinsic roles

Several studies have shown that β-catenin protein is overexpressed generally in AML blasts or cell lines versus levels in normal HSC [22,40–46] and associated with inferior patient survival [41,45,46]. Studies examining specific AML subtypes have demonstrated deregulated β-catenin and/or merit in targeting the molecule in normal karyotype [47], *FLT3* mutant [48], del(5q) [49], myelodysplastic syndrome (MDS) related [50], core binding factor (CBF) mutated [51], and PML-RARα^+^ [52] AML. Additionally, *in vivo* models of AML have shown β-catenin has a key role in the emergence, maintenance, and drug resistance of LSCs [53–59]; the cells responsible for the initiation and relapse of AML. In many of these studies the activation of β-catenin was insufficient to induce leukaemia as a single event but rather cooperated with well-known driver mutations including those involving the *mixed lineage leukaemia* (MLL) gene. *In vivo* models of MLL-induced AML seem to have particular dependence on β-catenin, but other studies have found β-catenin is dispensable for MLL leukemogenesis [35] which could be explained by Wnt signalling dose as above, or may depend on the cell of origin from which the AML has emerged [60].

### Cell extrinsic roles

In addition to its cell intrinsic role in leukaemogenesis, studies have also suggested β-catenin could exert some extrinsic influence over leukaemia development. In the normal bone marrow niche, targeted depletion of β-catenin in the stroma, results in diminished HSC maintenance and reconstituting capacity [61,62], whilst enforced β-catenin expression in the microenvironment enhanced HSC self-renewal and maintenance in a contact-dependent fashion [63]. Given the existence of this relationship in normal haematopoietic development, its perhaps unsurprising to see such an axis hijacked in leukaemia. Kode et al. reported that 38% of AML/MDS patients exhibited elevated β-catenin activity in osteoblasts, and targeted expression of an active β-catenin mutant in this cell type led to AML with recurrent genetic abnormalities [64]. Furthermore, the Le Beau group demonstrated that deletion of one copy of *Ctnnb1* was sufficient to prevent the MDS initiated in an *Apc* haploinsufficient microenvironment [50]. These results suggest an important role for dysregulated β-catenin in the microenvironment, but as before, complexity exists within the system, since targeted expression of a negative β-catenin regulator, DKK1, in osteoblasts has also been shown not to affect LSC homing or AML development [65].

That β-catenin is important at some point during AML initiation and progression seems well established; however, the molecular events leading to β-catenin stabilisation, mislocalisation and overactivity are less well understood.

## Mechanisms of β-catenin dysregulation in AML

The molecular aberrations leading to stabilised β-catenin in solid tumours such as colorectal, breast or liver cancer are well characterised. Mutations to Wnt signalling components such as APC, axin or β-catenin which constitutively activate the pathway are frequently observed and are fundamental drivers of these malignancies. However, such mutations are seldom reported in AML, or indeed any other haematological malignancy. Furthermore, we and others have shown that β-catenin mRNA is not particularly deregulated in AML, nor does it correlate well with β-catenin protein level [42,45,66], suggesting distinct post-transcriptional mechanisms must be active in AML (summarised in Table 1).

**Table 1:** Summary of mechanisms reported to dysregulate β-catenin in AML

Molecule	Mechanism of dysregulation	Outcome	References
γ-Catenin	γ-Catenin overexpression at the mRNA level functions to allow it to outcompete β-catenin for proteasomal degradation.	Promotes the stabilisation of β-catenin in AML cells. Degradation of β-catenin is prevented.	Muller-Tidow et al. [2004], Zheng et al. [2004], Tonks et al. [2007], Morgan et al. [2013], Qian et al. [2020]
GPR84	No definitive mechanism demonstrated, however GPR84 activates a Wnt transcriptional gene, as well as increased TCF4 expression. This may generate a positive feedback loop of Wnt activation.	Increase in the level and stabilisation of β-catenin in LSCs.	Dietrich et al. [2014]
FOXM1	May directly bind to β-catenin, preventing ubiquitination and degradation.	Increase in the level of β-catenin in MLL-rearranged AML LSCs.	Sheng et al. [2020], Zhang et al. [2011]
LEF-1	High LEF-1 may cause nuclear retention of β-catenin. LEF-1 may function as a chaperone to promote the entry of nuclear β-catenin.	Correlation between increased levels of LEF-1, and elevated nuclear β-catenin in AML blasts.	Morgan et al. [2019], Krieghoff et al. [2006], Behrens et al. [1996], Huber et al. [1996]
FLT3	Frequent receptor tyrosine kinase FLT3 mutations. Increased Y654 tyrosine phosphorylation of β-catenin.	Elevated levels of β-catenin activity within the nucleus.	Tickenbrock et al. [2005], Kajiguchi et al. [2007], Kajiguchi et al. [2012], Jiang et al. [2018]
α-Catenin	Frequent methylation of α-catenin promotes Wnt-mediated transcription, as there is no α-catenin present to negatively regulate β-catenin. β-catenin binding to TCF/LEF is no longer disrupted.	Increase in the nuclear activity of β-catenin.	Li et al. [2016], Qian et al. [2014], Chen et al. [2014], Ye et al. [2009]
PRL-3	Overexpression of PRL-3 stimulates dephosphorylation of Leo1 protein, increasing the affinity of Leo1 for β-catenin.	Increased prevalence of β-catenin within the nucleus, and heightened activation of Wnt target genes.	Chong et al. [2014], Chong et al. [2019]
WT1	Frequent overexpression and mutation.	Increase in nuclear β-catenin activity and Wnt transcriptional output.	Wagstaff et al. [in press]

### β-Catenin stabilisation

A prerequisite for the activation of β-catenin is the accumulation of this protein which must at some point exceed the rate of degradation mediated by the DC/proteasome. The heterogeneity of AML is reflected in the variety of mechanisms that have been purported to influence the stability of β-catenin in this disease.

The methylation of tumour suppressor genes which leads to their silencing and inactivation is a key driver of oncogenesis in AML and beyond. Numerous studies in both AML and MDS have reported the methylation of genes encoding secreted Wnt antagonists such as sFRP, WIF-1 and DKK [67–73]. These events would render normal or transformed HSPCs unable to restrict Wnt signals at the membrane leading to β-catenin accumulation.

Elsewhere, a number of recurrent chromosomal aberrations in AML such as t(8;21), t(15;17)[52], or t(9;11)[53–55] have been shown to increase β-catenin stability. In the case of CBF AMLs there have been frequent reports of γ-catenin (aka plakoglobin or *JUP*), a close structural and functional homolog of β-catenin, being up-regulated at the mRNA level [52,74,75]. Furthermore, subsequent functional studies have shown that γ-catenin overexpression can promote the stabilisation (and subsequent nuclear localisation and activity) of β-catenin in AML cells [42,76]. The most likely explanation for this is that the stabilised γ-catenin outcompetes β-catenin for the DC, thus saturating the complex, and allowing spared β-catenin to accumulate and participate in nuclear signalling activity. In t(9;11) or MLL-AF9 driven AML, GPR84, a G protein–coupled receptor, was shown to increase the total β-catenin protein level in LSCs without altering the inactive phosphorylated form [55]. Although no definitive mechanism for the β-catenin stabilisation was demonstrated, GPR84 did activate a Wnt transcriptional gene signature and increased TCF4 expression which could conceivably lead to positive feedback activation of Wnt components which stabilise β-catenin protein. More recently, FOXM1 has been shown to promote β-catenin stabilisation and nuclear import in MLL-rearranged AML [58], similar to previous reports in glioma [77]. Sheng and colleagues demonstrated that FOXM1 could directly bind β-catenin, protecting it from ubiquitination and degradation, and contributing to the FOXM1 mediated self-renewal and survival of MLL LSC.

Away from karyotypically abnormal AML, FLT3 mutations are highly prevalent in AML and have frequently been associated with the activation of Wnt/β-catenin signalling [48,78–80]. Work by Tickenbrock et al. showed that one potential mechanism by which this mediated was through induction of Frizzled-4 mRNA [78,81]; a Wnt receptor known to potentiate the pathway and ultimately lead to downstream β-catenin stabilisation. MDMX (a regulator of p53 stability) was recently shown to drive the progression of pre-LSC to overt AML [59]. Using both *PU.1* knockdown and *Tet2*-deficient *in vivo* preleukaemia models, the Steidl group showed MDMX could promote AML transformation through up-regulation of Wnt/β-catenin signalling in pre-LSC. Specifically, they demonstrated that MDMX could bind and reduce CK1 levels in the cell (a core DC component), thus leading to increased stability and nuclear accumulation of β-catenin.

### β-Catenin nuclear localisation and transcriptional activity

For β-catenin to elicit its aberrant transcriptional activity in AML, tight control of its nuclear localisation must be disrupted leading to disproportionately high levels of β-catenin protein in the nucleus of blasts and LSCs. The nuclear localisation of β-catenin is itself a poorly understood and heterogenous process [6], with several mechanisms also reported in AML.

Recently, we demonstrated that in addition to its role as a Wnt transcriptional partner, LEF-1 was also capable of modulating β-catenin nuclear levels in AML cells [43]. High LEF-1 correlated with elevated nuclear β-catenin in primary AML blasts, whilst ectopic LEF-1 could increase, and LEF-1 knockdown could reduce, nuclear β-catenin in AML cell lines. This could occur through LEF-1 serving a nuclear retention role for β-catenin as previously proposed [82], or alternatively could occur through LEF-1 serving as a chaperone to facilitate nuclear β-catenin entry as observed in other contexts [83,84].

β-Catenin is known to undergo several types of phosphorylation mediated by a plethora of kinases which govern stability, activity and distribution. Tyrosine phosphorylation of β-catenin (Y86, Y142, Y654) can release it from adhesion molecules such as E-cadherin and α-catenin on the inner membrane thus enhancing the pool of signalling competent β-catenin that can participate in transcription [85–88]. In chronic myeloid leukaemia (CML), tyrosine phosphorylation of β-catenin on residues Y86 and Y654 by the oncogenic fusion protein BCR-ABL led to its reduced affinity for the DC, and subsequent increase and activity in the nucleus [89]. In AML it has been shown that highly frequent mutations to the receptor tyrosine kinase FLT3 (internal tandem duplication; ITD and tyrosine kinase domain; TKD) increase Y654 phosphorylation of β-catenin leading to its enhanced abundance and activity in the nucleus [78–80]. There is an indication this likely represents hijacking of a normal mechanism since wild type (WT) unmutated FLT3 was capable of binding and tyrosine phosphorylating β-catenin [79]. Interestingly, a similar mechanism is driven by KIT, another frequently mutated receptor tyrosine kinase in AML, in mast cell leukaemia [90].

Related to above, the sequestration of β-catenin with adhesion molecules at the cell membrane is a key mechanism restricting it’s nuclear localisation and activity. In the AML cell line THP-1, the E-cadherin:β-catenin complex was found to be a key axis governing the capacity for monocytic differentiation in these cells, presumably by limiting transcriptionally active nuclear β-catenin [91]. α-Catenin is a close homolog of β-catenin that contains an NES and undergoes nucleocytoplasmic shuttling. In other tissues, it has been demonstrated as a negative regulator of β-catenin through disrupting its binding with TCF/LEF and inhibiting Wnt-mediated transcription [92–95], and also sequesters the molecule out of the nucleus back to membrane complexes [96]. In AML and MDS α-catenin is frequently methylated leading to loss of expression which is correlated with poor survival [97–100]. Although not formally demonstrated in a leukaemia context, one can envisage how the loss of α-catenin in AML may well increase the nuclear presence/activity of β-catenin, promoting leukaemogenic processes.

Phosphatase of regenerating liver-3 (PRL-3) is an oncoprotein implicated in many human cancers including AML, where it is overexpressed. Leo1 is a nuclear protein with roles in transcription, and a known downstream substrate of PRL3 [101]. In PRL-3 overexpressing AML cells it was demonstrated that PRL-3 induced dephosphorylation of Leo1 enhanced it’s interaction with β-catenin, retaining its presence in the nucleus and activating Wnt target genes [102]. Differential LEF-1 isoforms have been found to possess variable β-catenin binding capacity with N-terminally truncated isoforms lacking a β-catenin binding domain, whilst longer forms possess full β-catenin binding capability. A recent study from Feder et al. showed that whilst normal HSC predominantly expressed the short isoform, AML LSCs harboured preferential expression of the long LEF1 isoform thus priming them for β-catenin transcriptional activity and oncogenic gene expression [103]. Finally, we recently demonstrated that Wilms Tumor protein (WT1), a gene frequently overexpressed and mutated in AML [104], was capable of regulating nuclear β-catenin level and Wnt transcriptional output in AML cells (Wagstaff *et al*, in press). WT1 knockdown perturbed nuclear β-catenin localisation, whilst the conditional activation of commonly observed WT1 mutations in AML both stabilised β-catenin and augmented its signalling activity.

## Targeting β-catenin in AML

**Figure 2:**
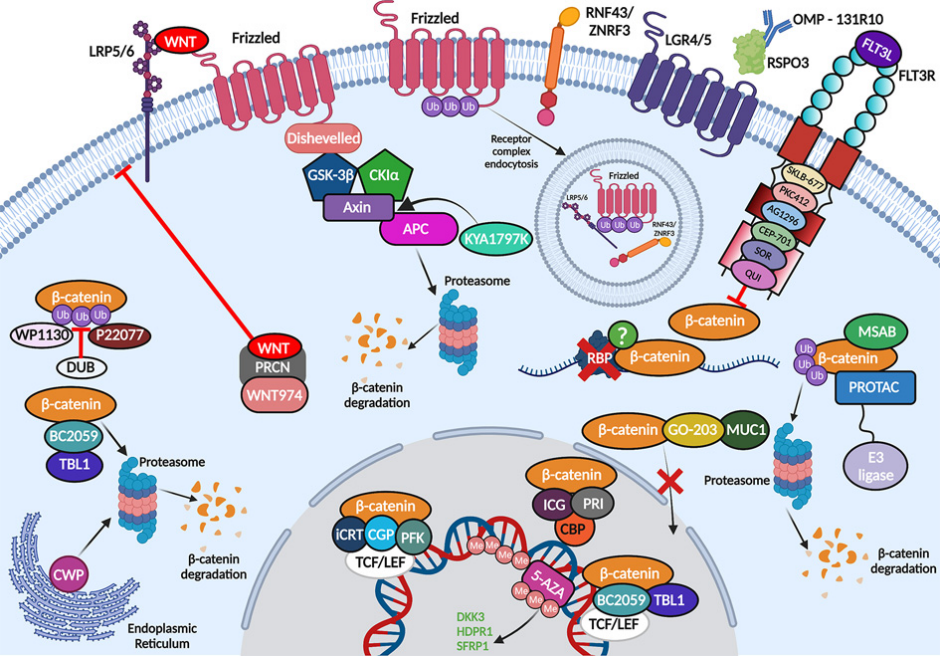
Pharmacological targeting of β-catenin AML Summary of the attempted and theoretical strategies used to therapeutically target oncogenic β-catenin stability an activity in AML cells. Compound abbreviations = CWP; CWP232291, CGP; CGP049090, PFK; PFK115-584, ICG; ICG-001, PRI; PRI-274, 5-AZA; 5-Aza-2′-deoxycytidine, PROTAC; Proteolysis-Targeting Chimaeras, MSAB; methyl 3-{[(4-methylphenyl)sulfonyl]amino} benzoate, SOR; Sorafenib, QUI; Quizartinib (created using Biorender).

Despite over 15 years’ worth of documented β-catenin overexpression, overactivity and mislocalisation in AML, an effective β-catenin inhibitor has not proceeded beyond clinical trial in this setting. Disconcertingly, aberrant β-catenin arising from *APC* mutations in colorectal cancer (CRC), a fundamental driver of intestinal tumourigenesis, has been known for even longer (early 1990s), yet β-catenin targeting agents are still absent in this disease. This might be explained by the fundamental differences between human and mouse biology which means *in vivo* findings have not been converted into clinical gain, or perhaps the adverse effects observed in healthy tissue through disrupting normal β-catenin function. Either way, attempts to target the molecule directly have proved challenging (superbly reviewed elsewhere by Cui et al. [105]) with most efforts thus far targeted at its transcriptional partners or upstream stabilising events (Figure 2).

### Targeting transcriptional partners

Given its transcriptional influence, it’s hardly surprising that many of the small molecular inhibitors (SMI) designed for β-catenin to date have targeted its interaction with the TCF/LEF factors or other transcriptional co-activators.

CGP049090 and PFK115-584 were among the first SMIs to be preclinically assessed in AML which prevent β-catenin:TCF/LEF complex formation and thus extinguish Wnt signalling output. These compounds delivered promising efficacy inducing apoptosis in both AML cell lines and primary blasts at submicromolar ranges whilst sparing normal peripheral blood mononuclear cells [106], a finding also replicated in chronic lymphocytic leukaemia (CLL) [107]. The iCRT (inhibitor of β-catenin responsive transcription) SMIs also inhibit β-catenin and TCF/LEF interaction, and iCRT3 has shown some promise in normal karyotype AML and acute lymphoblastic leukaemia (ALL) [108]. In the AML study, iCRT3 was effective in decreasing the expansion of co-cultured primary AML blasts, but exhibited mixed effects on the immunophenotype of these blasts (both pro-differentiation and pro-self-renewal) [47], in keeping with previous studies showing heterogenous sensitivity of AML blasts to β-catenin targeting [66].

β-Catenin also elicits its transcriptional activity through binding of the coactivators CBP and p300. Interaction with p300 has been shown to promote a differentiation phenotype, whilst interaction with CBP promotes a self-renewal output [109–111], an axis known to be active in normal HSC [112]. ICG-001 is an SMI that specifically binds the N-terminus of CBP, and not p300, disrupting interaction with β-catenin. In AML, positive results with ICG-001 have been observed in cells overexpressing PRL-3 (see above) when combined with the selective AKT/mTOR inhibitor [113], following success in ALL [114] and CML [115]. Although, ICG-001 appears not to have advanced into clinical trial for AML, a second generation CBP inhibitor PRI-724 (or C-82 prodrug [48]) has for advanced myeloid malignancies (NCT01606579), after showing promise in a solid tumour setting [116].

Transducin β-like 1 (TBL1) interacts with β-catenin to regulate both its stability, by preventing its ubiquitination and degradation [117], and its transcriptional activity by interacting with β-catenin to promote TCF/LEF-mediated transcription [118]. The Bhalla group showed that the anthraquinone oxime-analog BC2059 (Tegatrabetan) preferentially abolished the TBL1:β-catenin interaction which degraded β-catenin and abrogated Wnt target gene expression in AML cells [119]. This corresponded with increased apoptosis in cultured and primary AML cells *in vitro* and *in vivo*, and BC2059 also synergised with the pan-HDAC inhibitor panobinostat to kill AML cells. The same group has since shown that BC2059 can work synergistically with the JAK1/2 inhibitor ruxolitinib, or the BET protein degrader ARV-771, against secondary AML arising from post-myeloproliferative neoplasms, again by inhibiting β-catenin:TCF4 signalling [120].

### Targeting upstream Wnt signalling components

Given the absence of reported mutations to DC components or β-catenin itself in AML, which lead to constitutive β-catenin stabilisation, targeting events upstream of the Wnt signalosome may represent a viable strategy. Wnt ligand secretion from the cell first requires palmitoylation by the palmityl transferase Porcupine (PRCN) located in the endoplasmic reticulum [5]. Following encouraging data observed with inhibiting PRCN in CML [121] and solid tumours [122,123], Pepe and colleagues deployed the novel PRCN inhibitor WNT974 in AML cells [124]. WNT974 treatment decreased β-catenin target gene expression in primary AML samples and significantly diminished the *in vitro* replating efficiency of AML LSC (but not normal HSC) indicating that it could limit LSC self-renewal capacity without affecting apoptosis. Similarly, WNT974 reduced the *in vitro* replating capacity of CD117^+^ LSC derived from *Mll*^PTD/WT^*/Flt3*^ITD/WT^ mice (a potent *in vivo* model of AML [125]) but did not significantly impact engraftment or survival *in vivo* suggesting combinatorial approaches might be required.

As mentioned above loss of secreted Wnt antagonist expression is frequent in AML, primarily through methylation. Given the widespread epigenetic dysregulation observed in AML, the Tong group examined combinatorial approaches that could improve the efficacy of the methylation inhibitor 5-Aza-2′-deoxycytidine (decitabine, DAC) in AML treatment [126]. The authors found idarubicin (IDA) combined most affectively with DAC to induce apoptosis, and restrict the growth and proliferation, of AML cells *in vitro* and *in vivo*. Differential gene expression analyses revealed Wnt signalling as one of the major pathways perturbed by the regimen, and DAC/IDA successfully reversed *DKK3*, *HDPR1*, and *SFRP1* methylation in AML cell lines leading to reduced expression of β-catenin and its target genes.

R-Spondins are secreted Wnt agonists that potentiate the pathway through binding LGR4/5 [10,127] which sequester the transmembrane ligases RNF43/ZNRF3 away from Wnt receptors LRP5 and FZD preserving their presence on the membrane, which extends the intensity and duration of a Wnt signal [10]. This is a very well characterised mechanism in solid tissues but incompletely explored in a haematopoietic context. Once again utilising the MLL-AF9 or HOXA9/MEIS1 models of leukaemogenesis, known to be dependent on Wnt/β-catenin signalling (see above), Salik et al. demonstrated that the RSPO3-LGR4 axis could drive β-catenin signalling and leukaemogenicity *in vitro* and *in vivo* in these systems [128]. Satisfactorily, use of a clinical grade anti-RSPO3 monoclonal antibody (OMP-131R10; rosmantuzumab) markedly reduced the LSC content of primary AML patient samples, and PDX models, with negligible effects observed on the growth of normal HSC. Furthermore, anti-RSPO3 treated AML blasts exhibited enhanced differentiation and diminished engraftment potential. OMP-131R10 was found to reduce expression of LGR4 and HOXA9 which subsequently suppressed nuclear active β-catenin and several Wnt target genes implicated in self-renewal.

Finally, Hahn and colleagues presented data at the ASH 2019 annual meeting assessing the effectiveness of a novel β-catenin and RAS inhibitor KYA1797K. KYA1797K enhances β-catenin (and RAS) degradation through binding the RGS domain of axin and enhancing DC activity. It supressed the growth of multiple AML (and ALL) cell lines whilst preventing WNT3A-mediated activation of Wnt signalling through suppression of β-catenin and its target proteins [129].

### Targeting β-catenin stability indirectly

Other attempts to disrupt β-catenin stability in AML have targeted molecules outside of canonical Wnt signalling. Although it’s difficult to discern how much of their anti-leukaemia activity is through their target molecule versus Wnt signalling, it’s clear some of their activity is mediated through β-catenin inhibition.

As outlined above, WT or mutant FLT3 is capable of regulating β-catenin activity through modulating its stability, (tyrosine) phosphorylation status and localisation. Several studies employing a range of different FLT3 inhibitors such as SKLB-677 [130], PKC412, AG1296, CEP-701 [79], sorafenib and quizartinib [48], have shown them to be effective at targeting β-catenin activity/stability and/or AML LSCs whilst sparing normal HSC. Particular effectiveness of the latter compounds was observed when combined with inhibition of β-catenin (C-82) in *FLT3-ITD*^+^ AML cells [48].

Recently, a phase 1 clinical trial by the Cortes laboratory exploited the endoplasmic reticulum (ER) stress response to target β-catenin in relapsed AML and MDS [131]. CWP232291, and the active metabolite CWP232204, induce ER stress that leads to caspase activation and subsequent β-catenin degradation, which was effective against resistant prostate cancer [132]. The phase 1 study of CWP232291 in AML/MDS had acceptable toxicity, reduced survivin expression (β-catenin target gene), but had only minimal/modest overall efficacy indicating its deployment to eradicate Wnt signalling active LSCs may need combining with other agents [131].

MUC1-C expression is deregulated in AML blasts and LSCs and can increase leukemogenicity *in vivo* [133]. MUC1-C silencing reduced leukaemia initiating capacity which was mediated through reduced nuclear β-catenin translocation and suppressed survivin expression. The authors demonstrated that the MUC1-C inhibitor GO-203 could severely deplete active β-catenin expression and survivin expression, increasing the sensitivity of AML cells to cytarabine treatment.

## Novel avenues for β-catenin targeting in AML

Finally, there are some novel targeting strategies that have either not previously been deployed in AML or directed at β-catenin, which could represent desirable approaches to inhibiting the molecule in this setting. For a more extensive review of novel Wnt targeting strategies in cancer we direct the reader to an excellent review by Jung and Park [134], but this final section will focus on β-catenin targeting strategies that could have specific merit in AML (summarised with above in Figure 2).

### MSAB

Given that overexpression of β-catenin protein is primarily driven through post-transcriptional influences in AML cells, approaches which target direct degradation of the protein might have success in leukaemia. After performing a cell-based high throughput chemical screen of small molecules which disrupt TCF signalling output, the Lee group identified methyl 3-{[(4-methylphenyl)sulfonyl]amino} benzoate (MSAB) as a potent inhibitor of TCF reporter activity in HCT116 cells [135]. Further interrogation of the mechanism revealed that MSAB could bind β-catenin in its armadillo region and target the molecule for direct proteasomal degradation thus limiting its nuclear presence and activity. This compound suppressed the growth of several Wnt-dependent cell lines that were almost exclusively of epithelial origin, but no haematological cells were tested.

### PROTAC

PROteolysis-TArgeting Chimaeras (PROTACs) have emerged as a promising strategy for degrading a protein of interest (POI) and have already been trialled for β-catenin in alternative settings. PROTACs are heterobifunctional molecules consisting of a POI ligand chemically linked to an E3 ligase ligand. Upon capture of the target protein by the PROTAC, the closely associated E3 ligase polyubiquitinates the POI stimulating its degradation in the proteasome [136]. Liao et al. constructed a β-catenin targeted PROTAC exploiting the axin binding domain (amino acids 469–482) with two stapled peptides (SAHPA1 and xStAx) linked to the VHL recognition peptide sequence ALAPYIP, widely employed as a ligand for the VHL E3 ligase [137]. This agent was effective at degrading both endogenous and WNT3A induced β-catenin, as well as reducing Wnt signalling output in HEK293T and various CRC cell lines. The PROTAC also reduced tumour formation and growth in both xenograft and *APC^min/+^* mouse models. Provided PROTACs can permeate the membranes of myeloid cells with similar efficiency, the employment of PROTACs seems an entirely sensible strategy to reducing the excessive β-catenin protein levels observed in AML cells.

### DUB inhibition

Similarly, along the ubiquitination theme, deubiquitinases (DUBs) work through antagonising the actions of E3 ligases by removing ubiquitin groups from target proteins, thus promoting their stability. DUBs such as USP2A [138], USP4 [139], USP9X [140,141] and USP20 [142] have emerged as critical regulators of β-catenin stability and Wnt signalling output, but will likely have tissue and developmental restricted expression. Novel DUB inhibitors such as WP1130 (degrasyn) have anti-cancer action [143], and have been deployed effectively in haematological malignancies such as T-ALL [144] and AML [145], albeit β-catenin modulation was not a characterised mode of action. In particular, disrupting USP7 activity might represent a direct β-catenin-targeting mechanism in AML given USP7’s known regulation of β-catenin stability [146,147], and recent evidence demonstrating that USP7 inhibition (both genetic; siRNA, and pharmacological; P22077) reduces AML cell proliferation and enhances chemosensitivity [148].

### RBP networks

**Figure 3:**
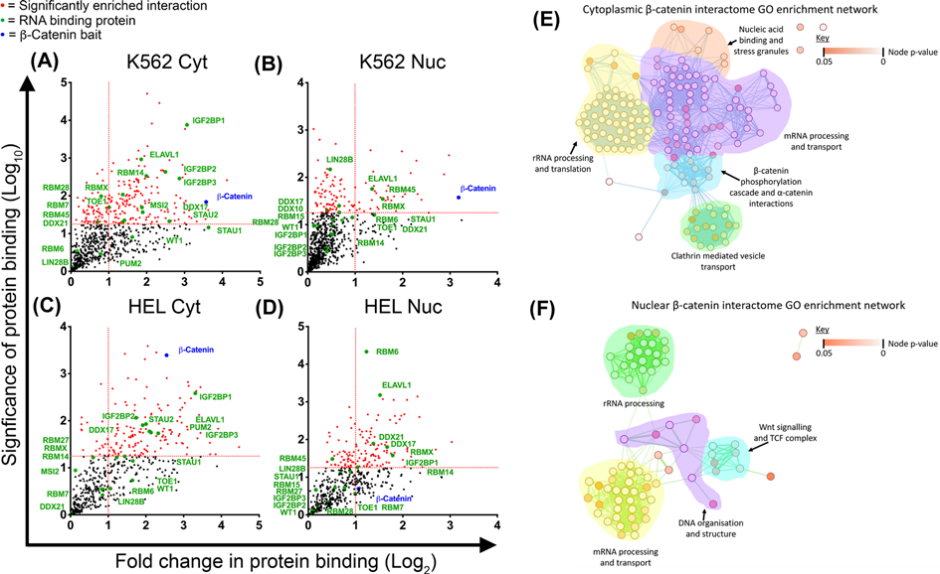
β-Catenin interactions with RBPs in myeloid cells Scatter plots showing RBP interactions detected in β-catenin interactomes performed in (**A**) K562 cytosolic, (**B**) K562 nuclear, (**C**) HEL cytosolic and (**D**) HEL nuclear fractions. Vertical dashed red line indicates the threshold for 2-fold change in protein binding at log_2_ (= 1) relative to IgG co-IP. Horizontal red line represents threshold for significant interactions at *P*=0.05 on log_10_ scale (= 1.3). Highlighted red dots indicate statistically significant interactions and green labels indicate RBPs. Combined GO term network demonstrating biological processes of β-catenin interacting proteins found in the (**E**) cytoplasmic and (**F**) nuclear fractions of HEL, HL60 and K562 cells. Nodes (circles) in the network represent GO terms associated with the β-catenin interacting proteins. Red nodes represent GO terms with relatively high adjusted enrichment *P*-values (i.e., *P*-value is close to 0.05 which indicates term is less accurate at representing a function or process associated with the gene list). Comparatively, white nodes indicate GO terms with lower *P*-values close to 0. Edges (lines between nodes) representing how closely related the GO terms are to one another, with thick lines representing closely related GO terms.

Finally, our group recently performed the first β-catenin interactome in myeloid cells, representing the first exploration of β-catenin protein partners in haematopoietic cells of any kind [43]. From this we noted the significant enrichment of RNA-binding proteins (RBP; Figure 3A–D) which are key molecules regulating various facets of RNA biology including biogenesis, splicing, stability, processing, transport and translation [149]. RBPs are rapidly emerging as critical regulators of normal haematopoietic development and are heavily implicated in leukaemia [150]. β-Catenin’s association with RBPs, particularly those related to ‘mRNA processing and transport’ (Figure 3E,F), suggest a previously undefined role for β-catenin in regulating RNA biology in AML. In epithelial tissue β-catenin has been shown to bind RBPs and regulate pre-mRNA splicing [151], whilst in vascular smooth muscle cells it can regulate protein translation [152]. Furthermore in CRC cells, β-catenin can even bind certain oncogenic mRNAs directly such as COX-2 and CyclinD1 through AU-rich elements of 3’-UTRs, promoting their stabilisation [153–156]. Should β-catenin perform such a role in AML cells, it may well explain why pharmacological attempts to disrupt β-catenin interaction with transcriptional partners (e.g. TCF/LEF) has not yielded the clinical success expected, because β-catenin may also influence post-transcriptional expression. Therefore, future attempts to totally extinguish aberrant β-catenin activity in AML may require a two-pronged attack to limit both its transcriptional and post-transcriptional influences on gene expression.

## Conclusion

The rationale for exploring Wnt signalling therapeutics in AML is both sound and justified, however β-catenin has proved a challenging component to pharmacologically target in this setting. This challenge is a reflection of the genetic heterogeneity of AML where a multitude of mechanisms have been proposed to dysregulate the molecule in blasts and LSCs. These mechanisms are more diverse, elusive, and intricate than the relatively simple genetic mutations that occur to single Wnt signalling components (e.g., Axin or APC) in solid cancers. Only the full and thorough characterisation of these mechanisms in AML cells would inform effective drug design; however, this may be an impractical task given the considerable inter-tumour heterogeneity displayed between AML patients. In which case, for now the continued development of novel agents that bind and directly destabilise the excessive β-catenin protein in AML cells holds the most promise, since only minimal levels/activity of the protein are required to sustain the maintenance and development of normal HSC. The ongoing molecular characterisation of β-catenin in a haematopoietic context will be vital in revealing new vulnerabilities that can be exploited through combinatorial approaches to extinguish β-catenin activity in AML.

## Open Access

Open access for this article was enabled by the participation of University of Sussex in an all-inclusive *Read & Publish* agreement with Portland Press and the Biochemical Society under a transformative agreement with JISC.

## Abbreviations

AML: acute myeloid leukaemia
CLL: chronic lymphocytic leukaemia
DUB: deubiquitinase
POI: protein of interest
RBP: RNA-binding proteins
SMI: small molecular inhibitors
TBL1: transducin β-like 1
WT: Wild Type
WT1: Wilms Tumor protein
